# Criticising the Journal of the American Medical Association

**Published:** 1884-02

**Authors:** 


"CRITICISING THE JOURNAL OF THE AMERICAN MEDICAL ASSOCIATION."

Several of the smaller medical periodicals in this country have thought it proper to call attention in a public manner to the columns of the *Journal of the American Medical Association*, which have exhibited in the past a number of blunders, some trivial, some gross enough to make one blush for the knowledge which printers and proof-readers display of even the common words in our mother tongue. The *New York Medical Record* of December 22d, 1883, charitably declines to publish a letter from a Connecticut correspondent severely commenting on the same facts.

We should not have spoken upon this point if the editor himself had not thought fit to make a semi-humorous response to one of his critics. What we have to say may be set down in very few words. We have acutely suffered, as what editors have not, from errors apparently of the very grossest ignorance, for which we were not in any way responsible. The *Journal of the American Medical Association* is in a position to command the respectful consideration of every fair-minded member of the medical profession in this country. It has just completed the first year of issue, and if its young critics will take the pains to look over the first year of such a journal, for example, as the *London Lancet*, and amidst its typographical and grammatical errors, peruse the accounts which it contains of the movements of the British court, and the latest theatrical representations, we think he will look more leniently on the facts in the present case.

The *Journal of the American Medical Association* is not the property of its veteran editor. It belongs to the American Medical Association. It does not appertain to a large publishing house, nor to a medical school, nor to a wholesale drug manufacturing firm, nor to a man with an enemy to injure or a scheme to promote. It belongs to the profession of the country; and, being in this respect quite singular, we entertain for it a feeling of respect and admiration, which several dozen more typographical errors could not destroy. We call on every man in the country who would like to see the profession represented one day by a thoroughly independent publication of its own, fearless of every crusted abuse and incorruptible by the wealth of any corporation, to support this journal by financial and moral aid. It is in its first and weakest stage. It is as sure to become in the future what its best friends desire it should be, as the members of the profession it represents are sure to advance in the cultivation of science.

We beg its critics to wait; and we beg also its patient editor to refrain from answering them. We look to see its columns present each day more of a national and less of a local character; and we hope to see it before long repeating the utterances of the very best men in every part of the country.

---

THE *Universal Bibliographic Review of the Medical Sciences*, with an annual alphabetical index indicating the articles contained in special journals, and the works published in every language and in every country, methodically classified according to the subjects discussed, followed by an alphabetical list of authors, is announced as a monthly publication about to appear under the management of Dr. Ote. Meyners D'Estray.

The object of this review is to enable the practitioner and the author to turn at once to the sources which they desire to consult upon any subject whatever.

The *Bibliographic Review* will form every year a handsome octavo volume of at least 600 pages. The price of subscription is 30 francs annually. In order to subscribe, address M. B. Grémiaux, General Secretary, Place Saint-Michel, 6, Paris.

NUMBER OF BIRTHS IN FRANCE.

During last year there were 970,843 births, of which 961,203 were simple, that is, there was birth of a single child; 9,333 were twins, and 107 were triplets. Among the simple births, 829,440 were born alive, and 40,763 were dead born. Of multiple births, 205 infants were born alive, 116 dead. Therefore, it can be seen that the twin births gave about three times more dead born than the simple births, and that among the multiple births the proportion is nine times greater.—(*Le Praticien.*)

